# Causal association between placental growth factor and coronary heart disease: a Mendelian randomization study

**DOI:** 10.18632/aging.205061

**Published:** 2023-10-02

**Authors:** Bo Zuo, Sha Zhu, Guoting Zhong, Haoyang Bu, Hui Chen

**Affiliations:** 1 Department of Cardiology, Cardiovascular Centre, Beijing Friendship Hospital, Capital Medical University, Beijing 100050, China; 2 Department of Neurology, Peking University International Hospital, Beijing 102206, China; 3 Department of Neurology, The First Hospital of Handan, Handan, China

**Keywords:** coronary heart disease, myocardial infarction, placental growth factor, Mendelian randomization, causal association

## Abstract

Objective: Placental growth factor (PlGF), an important polypeptide hormone, plays an important regulatory role in various physiological processes. Observational studies have shown that PlGF is associated with the risk of coronary heart disease (CHD). However, the causal association between PlGF and CHD is unclear at present. This study aimed to investigate the causal association between genetically predicted PlGF levels and CHD.

Methods: Single nucleotide polymorphisms (SNPs) associated with PlGF were selected as instrumental variables (IVs) to evaluate the causal association between genetically predicted circulating PlGF levels and CHD risk by two-sample Mendelian randomization (MR).

Results: Inverse variance weighted (IVW) analysis showed that there was a suggestive causal association between genetically predicted PlGF level and the risk of CHD (OR = 0.79, 95% CI: 0.66–0.95, *P* = 0.011) overall. In addition, PlGF levels had a significant negative causal association with the risk of myocardial infarction (OR = 0.83, 95% CI: 0.72–0.95, *P* = 0.007). A negative correlation trend was found between PlGF level and the risk of angina pectoris (OR = 0.89, 95% CI: 0.79–1.01, *P* = 0.067). In addition, PlGF levels had a significant negative association with the risk of unstable angina pectoris (OR = 0.78, 95% CI: 0.64–0.94, *P* = 0.008). PlGF levels were negatively correlated with CHD events with suggestive significance (OR = 0.89, 95% CI: 0.80–0.99, *P* = 0.046).

Conclusion: Genetically predicted circulating PlGF levels are causally associated with the risk of CHD, especially acute coronary syndrome, and PlGF is a potential therapeutic target for CHD.

## INTRODUCTION

Coronary heart disease (CHD) remains the leading cause of death worldwide [1] and is characterized by the formation of coronary atherosclerotic plaques, causing coronary artery stenosis and finally leading to episodic or persistent angina pectoris (AP). Plaques are mainly composed of lipids, calcium, and inflammatory cells. When plaque rupture results in thrombosis, it can cause myocardial infarction (MI) and even death in severe cases. Although great progress has been made in the treatment of CHD, including reperfusion therapy such as percutaneous coronary intervention (PCI) and secondary prevention treatment of anti-platelet, lipid-lowering, management of hypertension, diabetes and other risk factors, its pathogenic factors and pathophysiology are still not completely clear, and the prevention and treatment situation is still very serious. Therefore, it is of great practical significance to search for effective therapeutic targets for CHD and improve cardioprotection strategies.

Placental growth factor (PlGF) is a member of the VEGF family, which is mainly expressed in placenta, heart and lung tissues [2]. PlGF is a polypeptide hormone with various physiological effects and is involved in the immune response, vascular homeostasis, angiogenesis and other physiological activities, and it plays a key role in cellular metabolic activities [3]. Studies have shown that PlGF can promote angiogenesis, growth and survival of endothelial cells [4, 5]. It can also promote vascular inflammation by activating the expression of adhesion molecules and chemokines in endothelial cells, which plays key roles in the occurrence and development of cardiovascular diseases, including CHD [6].

Clinical studies have shown that PlGF is elevated quickly in myocardial infarcted tissue of patients with MI, and the serum PlGF level is positively correlated with the improvement of left ventricular function [7]. Signs of vascular ageing occurred in women with low PlGF levels in the second trimester of pregnancy in the next 6 to 9 years, supporting that PlGF is required for maintaining normal cardiovascular function [8]. However, it has also been found that PlGF, released by heart tissue during ischaemia [9], is associated with mortality after acute coronary events and has potential value in predicting mortality after acute coronary syndrome (ACS) [10, 11]. In addition, a high baseline plasma PlGF level was associated with an increased risk of cardiovascular death, MI, and stroke, but these associations disappeared or attenuated after adjusting for known cardiovascular risk factors [12]. PlGF may be a long-term biomarker for the risk of coronary heart disease. There is a moderate correlation between plasma PlGF levels and the risk of coronary heart disease in women, and PlGF levels can predict myocardial infarction events several years in advance [1].

**Figure 1:**
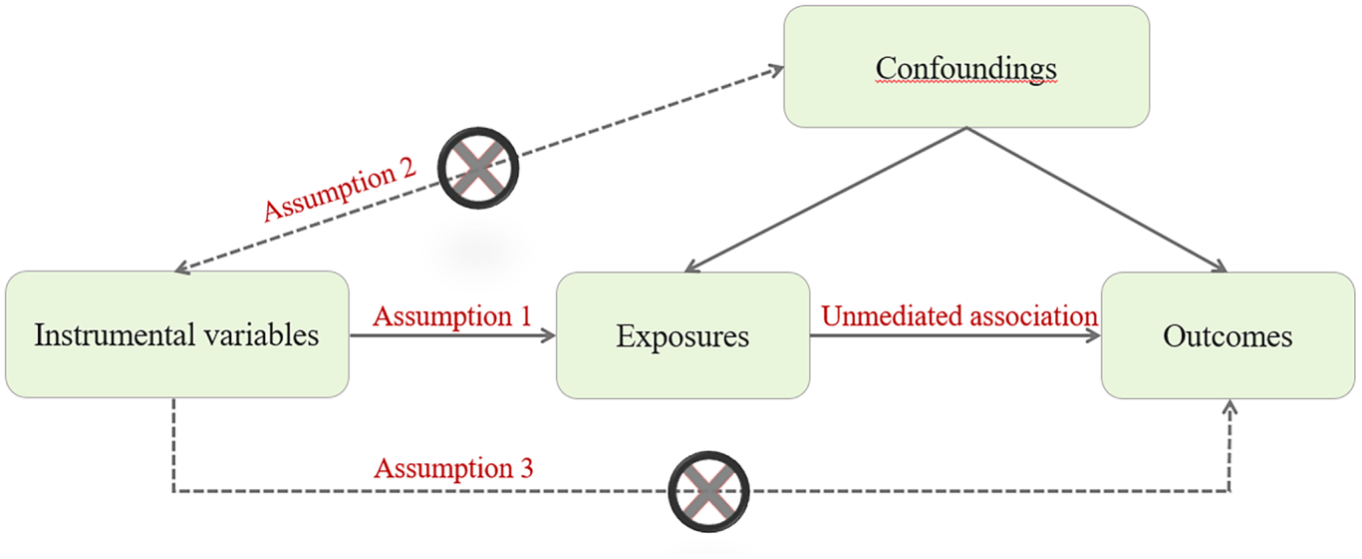
MR assumptions for a two-sample MR study.

The causal association between PlGF level and the risk of CHD is not clear, as traditional observational studies are prone to bias due to residual confounding effects and reverse causality. Therefore, it is necessary to conduct a Mendelian randomization (MR) study to evaluate the causal association between PlGF level and the risk of CHD. The basic principle of MR design is that genetic variation is fixed at conception and randomly assigned to individuals. MR must meet the following three basic principles: (1) the instrumental variables must be associated with the exposure factors to be studied; (2) Instrumental variables must be independent of confounding factors; (3) Instrumental variables can only be related to the outcome by influencing the exposure factors to be studied. Therefore, MR design can be conceptualized as a natural experiment and overcome the limitations of traditional observational studies (Figure 1) [13, 14]. Therefore, this study aimed to investigate the potential causal association between PlGF levels and CHD using MR analyses.

## RESULTS

### MR analyses

There was a suggestive causal association between the genetically predicted PlGF level and the risk of CHD overall. The higher the PlGF level was, the lower the risk of CHD (OR = 0.79, 95% CI: 0.66–0.95, *P* = 0.011).

**Figure 2:**
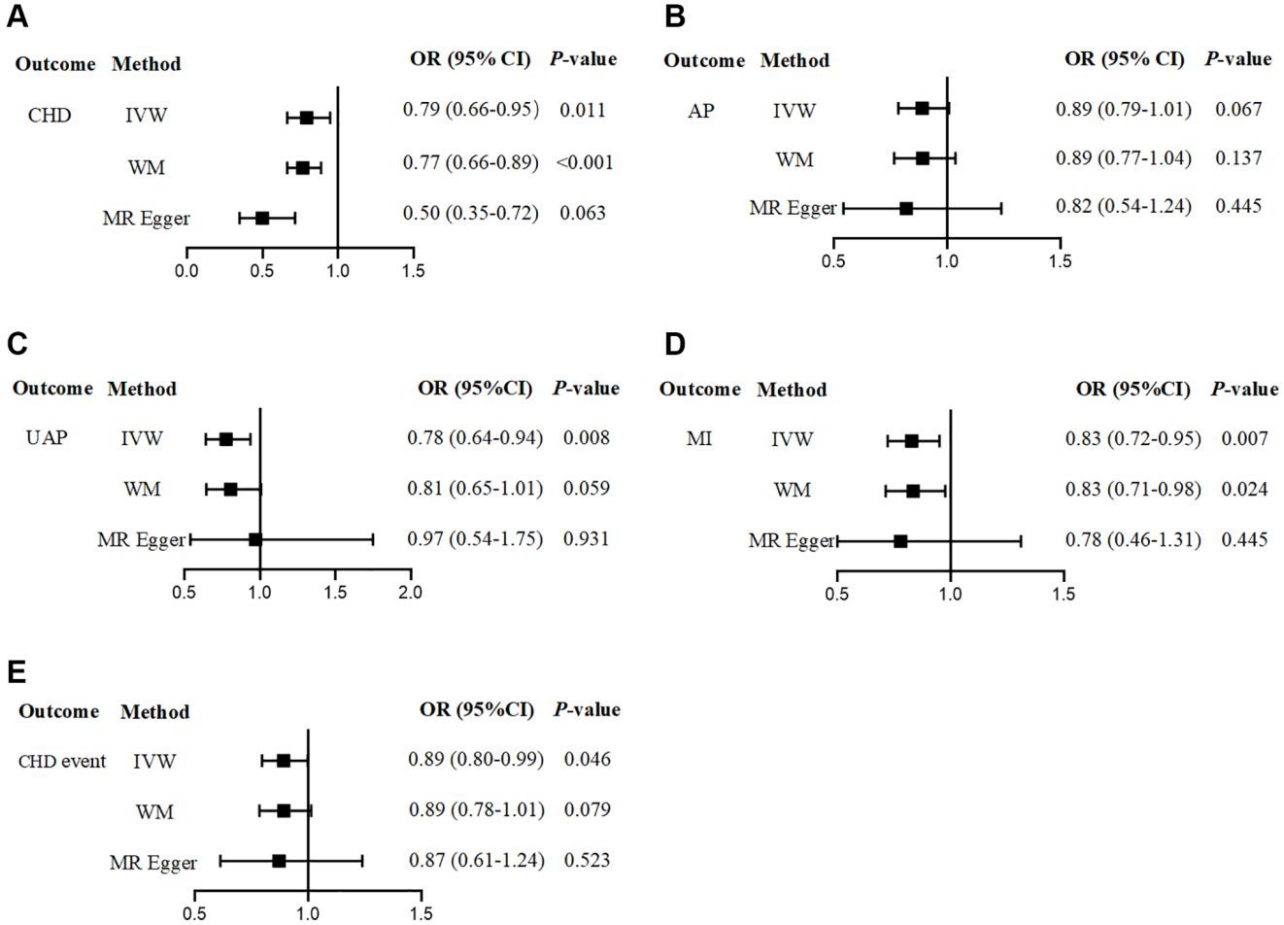
**The effect of genetically determined circulating PlGF level on the risk of CHD.** (**A**) CHD; (**B**) AP; (**C**) UAP; (**D**) MI; (**E**) CHD event. Abbreviations: CHD: coronary artery disease; AP: angina pectoris; UAP: unstable angina pectoris; MI: myocardial infarction.

Subsequently, we also analysed the CHD subgroup. We observed that there was no statistical association between the genetically predicted PlGF level and the risk of AP, but there was still a negative correlation trend (OR = 0.89, 95% CI: 0.79–1.01, *P* = 0.067). There was a significant negative association between the genetically predicted PlGF level and the risk of UAP (OR = 0.78, 95% CI: 0.64–0.94, *P* = 0.008). We found that there was a significant causal association between the genetically predicted PlGF level and the risk of MI. The higher the PlGF level was, the lower the risk of MI (OR = 0.83, 95% CI: 0.72–0.95, *P* = 0.007). In addition, a suggestive negative association was found between the genetically predicted PlGF level and the adverse events of CHD (OR = 0.89, 95% CI: 0.80–0.99, *P* = 0.046) (Figure 2, Supplementary Table 1). The scatter plot shows the impact of circulating PlGF levels on the risk of different types of CHD (Supplementary Figure 1).

### Sensitivity analysis

As shown in Table 1, in all MR analyses in this study, Cochran’s *Q* statistics did not find significant heterogeneity, and MR Egger regression analysis did not find evidence of significant pleiotropy. The estimated values obtained after removing a single SNP had no significant change in the leave-one-out test, indicating that no single SNP had a significant effect on the overall estimation (Supplementary Figure 2).

**Table 1:** Heterogeneity test and horizontal pleiotropy test of PlGF associated SNPs.

**Outcome**	**Heterogeneity test**				**Horizontal pleiotropy test**	
	**IVW Cochran’s *Q***	**IVW P**	**MR-Egger Cochran’s *Q***	**MR-Egger P**	**Intercept**	**SE**	**P for intercept**
CHD	7.179	0.066	0.114	0.944	0.04	0.015	0.117
AP	2.429	0.488	2.241	0.326	0.007	0.017	0.721
UAP	0.693	0.875	0.07	0.966	−0.019	0.024	0.513
MI	3.275	0.351	3.18	0.204	0.005	0.021	0.829
CHD event	1.286	0.732	1.268	0.530	0.002	0.014	0.906

Abbreviations: PlGF: placental growth factor; IVW: inverse variance weighted; CHD: coronary artery disease; AP: angina pectoris; UAP: unstable angina pectoris; MI: myocardial infarction; SE: standard error.

## DISCUSSION

In this study, we investigated the association between the genetically predicted level of PlGF and the risk of CHD. Overall, there is a suggestive, negative causal association between the PlGF level and the risk of CHD. A significant negative causal association was found between the PlGF level and the risk of MI and UAP. A suggestive negative association exists between the level of PlGF and the adverse events of CHD. The PlGF level was not statistically related to the risk of AP, but there was still a trend towards a negative association. These results indicate that PlGF may be a protective factor for cardiovascular disease.

Clinical studies have shown that PlGF may be a long-term biomarker for the risk of coronary heart disease. There is a moderate correlation between plasma PlGF levels and the risk of coronary heart disease in women, and PlGF levels can predict myocardial infarction events several years in advance [15]. The increase of PlGF is a new independent predictor of incidence rate and mortality of long-term cardiovascular disease in patients with Type 1 diabetes nephropathy [16]. Lenderink et al. found that elevated plasma levels of PlGF are associated with adverse cardiac outcomes during long-term follow-up (a median follow-up period of four years) in ACS patients [17]. Bui et al. also reported that higher concentration of PlGF is associated with long-term risk of recurrent cardiovascular events independent of traditional risk factors in ACS patients [18]. In addition, PIGF may also be a short-term prognostic biomarker of coronary heart disease risk. Elevated PlGF concentration has become an important independent biomarker for short-term adverse outcomes in patients with acute chest pain and known or suspected ACS [19].

Oxidative stress and apoptosis play key roles in myocardial ischaemia-reperfusion injury [20, 21]. PlGF is a selective ligand of VEGFR1 (vascular endothelial growth factor receptor 1). In heterozygous VEGFR1 knockout mice, the protective effect of ischaemic preconditioning on the heart was significantly inhibited [20, 22], suggesting that VEGFR1 plays an important role in cardioprotection. Zhang et al. found that pretreatment with PlGF could significantly improve ischaemia/reperfusion injury, reduce the infarct area, improve cardiac function, and reduce the degree of cardiomyocyte apoptosis using a mouse heart ischaemia/reperfusion model. They also confirmed that PlGF can inhibit the production of reactive oxygen species (ROS) in the mitochondria of cardiomyocytes after the activation of VEGFR1. Further studies showed that pretreatment with PlGF could activate the phosphorylation of Akt and GSK-3β and inhibit the activation of caspase-3 after reperfusion [23]. This shows that PlGF can activate VEGFR1 to protect the heart from ischaemia-reperfusion injury by inhibiting oxidative stress and reducing cardiomyocyte apoptosis.

Previous studies have shown that PlGF is beneficial to angiogenesis and arteriogenesis of ischaemic myocardium [3, 24, 25]. PlGF can promote the proliferation of endothelial cells and recruit bone marrow cells to target tissues [24]. As a selective ligand of VEGFR1, PlGF can activate VEGFR1 to promote angiogenesis and endothelial cell growth without any related side effects, such as oedema, hypotension and the occurrence of haemangioma [24, 25]. Takeda et al. found that exogenous recombinant PlGF treatment not only improved the survival rate after MI and improved cardiac function but also significantly increased the number of CD31-positive cells and α-smooth muscle actin-positive vessels in the infarcted area and mobilized endothelial progenitor cells into the peripheral circulation [26]. The above research shows that PlGF can reduce the infarct area after MI and improve cardiac function by enhancing angiogenesis and arteriogenesis.

Studies have shown that PlGF may also have a direct protective effect on cardiomyocytes [26–28]. Roncal et al. found that exogenous PlGF treatment can induce compensatory hypertrophy of cardiomyocytes in noninfarcted myocardium and improve cardiac recovery after MI by using a mouse MI model [28]. PlGF also promotes angiogenesis at the infarct border and vascular dilatation in the distal myocardium, increasing the vascular perfusion area and improving the adaptive remodelling of the heart after MI [28]. These results suggest that PlGF can directly improve cardiac function and promote adaptive remodelling after MI.

In addition, some studies have shown that PlGF is proatherogenic, attributed to its ability to activate endothelial adhesion molecule expression and monocyte recruitment to the arterial wall [6, 29, 30]. However, experimental studies have not found any evidence for a pathogenic role of PlGF in more advanced stages of atherosclerosis [31]. In contrast, PlGF may be beneficial to maintain the stability of advanced atherosclerotic plaques because it could stimulate endothelial cell proliferation [32], which provides an important basis for the protective role of PlGF in CHD.

This study explored the causal association between PlGF levels and different types of CHD, including AP, UAP, MI and CHD events. The effect estimates of different data sources all point to the same direction, further strengthening the negative causal association between genetically predicted PlGF level and CHD risk. In particular, the PlGF level was significantly associated with ACS, including UAP and MI, which suggests that PlGF may be a potential predictor and potential effective target of ACS, and its mechanism is worthy of further investigation. Sensitivity analysis was also carried out in this paper, and the trend of the results did not change, thus increasing the reliability of this study.

This study also has some limitations. First, the number of SNPs of IV used in this study was relatively small, which limited the power to detect associations. Second, the data of this study are all from European populations. Although it reduces the deviation caused by population stratification, further testing is needed to determine whether it can be extended to other populations. Third, the available data in the study are summary statistics, without data at the individual level. Therefore, it may bring inevitable deviation to the study. Fourth, in the leave-one-out method, several SNPs removed, the influence of the remaining SNPs on the outcome was inconsistent with the overall outcome, and the data was biased.

In conclusion, this study used a two-sample MR method to explore the causal association between genetically predicted PlGF levels and CHD. The results showed that the genetically predicted PlGF level was negatively correlated with the risk of CHD, especially ACS. PlGF is a potential effective target for the prevention or treatment of CHD. Future research needs to explore the mechanism of PlGF in the occurrence and development of CHD, especially ACS.

## MATERIALS AND METHODS

### Study design

**Figure 3:**
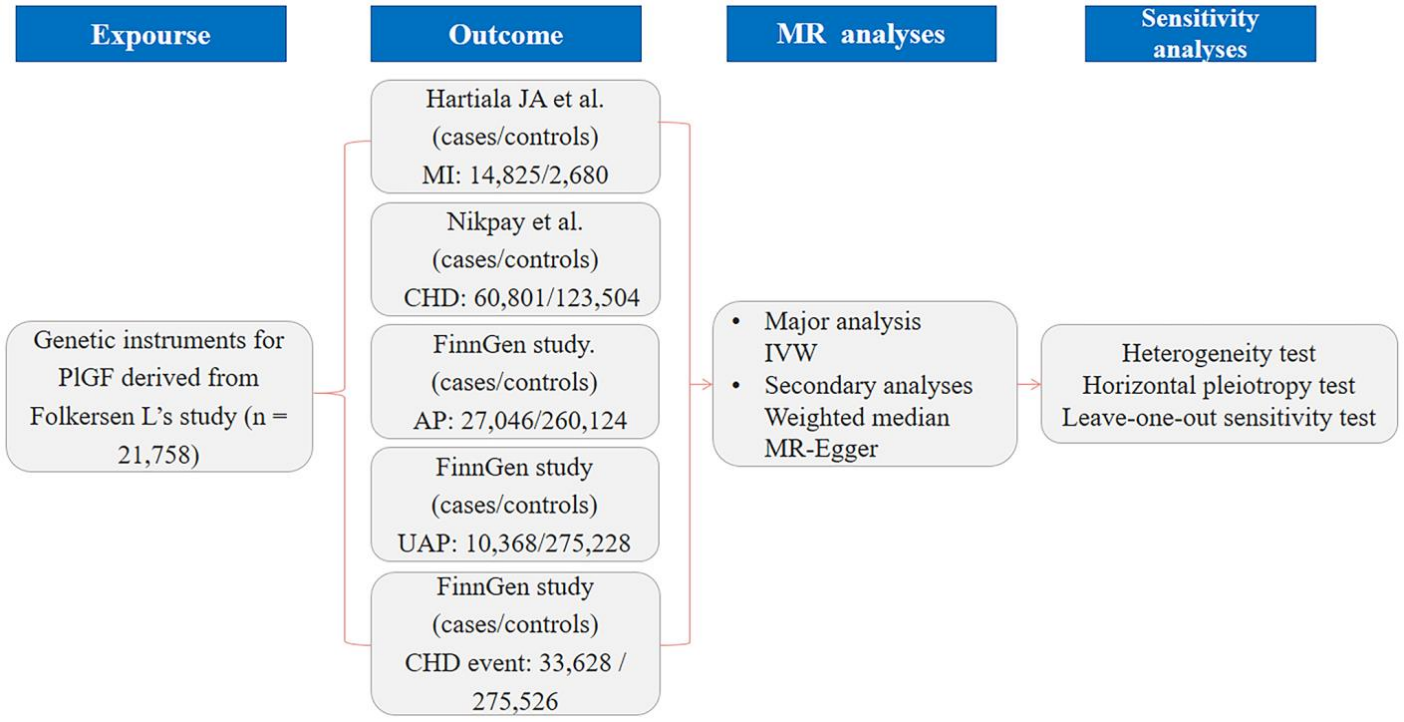
**Flowchart of the study design overview.** Abbreviations: PlGF: placental growth factor; CHD: coronary artery disease; AP: angina pectoris; UAP: unstable angina pectoris; MI: myocardial infarction; IVW: inverse variance weighted; MR: Mendelian randomization.

We performed 2-sample MR analyses to evaluate the causal association between PlGF and CHD. All data were obtained from the currently published genome-wide association study (GWAS) and Finn Gen consortium. Additional ethical approval or informed consent was not needed, as ethical informed consent and approval were completed in the original studies. The flowchart of the study design overview is shown in Figure 3. Single nucleotide polymorphisms (SNPs) associated with PlGF were selected as genetic instrumental variables (IVs). Pooled GWAS statistics of outcomes related to CHD, MI, AP, unstable angina pectoris (UAP) and major CHD events were selected from published large-scale GWAS meta-analyses and the FinnGen consortium. Genetic outcome associations were extracted and analysed by MR analyses, and corresponding sensitivity analyses were performed.

### Selection of instrumental variables

The genome-wide significant SNPs (*P* < 5 × 10^−7^) after linkage disequilibrium clustering were selected from the study by Folkersen et al. as the instrumental variable of PlGF for this 2-sample MR study. The study included 30931 participants of predominantly European descent and mapped and replicated protein quantitative trait loci (pQTL) for 90 cardiovascular proteins, resulting in 451 pQTLs for 85 proteins [33]. Linkage disequilibrium among all SNPs was performed based on the European 1000 Genome Project reference panel. The independent SNP was selected by clumping (r^2^ ≤ 0.001), and the SNP with the smallest *P* value was retained. The SNP characteristics related to PlGF are shown in Table 2, Supplementary Table 2.

**Table 2:** The characteristics of the selected PlGF associated SNPs.

**SNP ID**	**Chr**	**Position**	**EA**	**EAF**	**Beta**	**SE**	***P***
rs184474	1	7954463	G	0.3974	0.0538	0.0106	3.93E-07
rs10182686	2	40610830	G	0.4541	−0.0517	0.0103	4.76E-07
rs9551468	13	28985316	G	0.5227	0.0835	0.0102	2.97E-16
rs175510	14	75524839	A	0.4587	0.1149	0.0102	1.27E-29

Abbreviations: PlGF: placental growth factor; SNP: Single nucleotide polymorphism; EA: effect _ allele; EAF: effect allele frequency; Chr: chromosome; SE: standard error.

### Outcome GWAS dataset selection

The participants of the outcome-related GWASs selected in this study were mainly of European descent. The summary data for CHD were obtained from a large GWAS that conducted a meta-analysis of 48 studies, collecting 60801 cases and 123504 controls [34]. The summary data for MI came from a large GWAS that conducted a meta-analysis of 48 studies, collecting 61000 cases and 577000 controls [35]. The outcome summary data on AP, UAP, and major CHD events were obtained from the FinnGen consortium (data freeze 7). For the definition of major CHD events, please refer to Supplementary Table 3 and the website: https://risteys.finregistry.fi/endpoints/I9_CHD. Among them, 27046 cases and 260124 controls were collected from the summary data of AP, and 10368 cases and 275228 controls were collected from the data of UAP. The major CHD adverse events were collected in 33628 cases and 275526 controls. Relevant participants and statistical analysis, gene platform for detailed information, please visit FinnGen website (https://www.finngen.fi/en/).

### Data extraction and MR analysis

Two-sample MR analysis should be performed according to the identified exposure factor SNPS, and the information of SNPS in the outcome should be extracted. We extracted gene-outcome association information from the corresponding outcome of GWAS data using the four SNPs identified by the exposure instrumental variables. All MR analyses were performed using the “Two Sample MR” package in R software (version 4.1.2 with packages, R Foundation for Statistical Computing, Vienna, Austria). To ensure the validity of our conclusions, we used Bonferroni correction for *P* values in the primary analysis with a threshold of *P* < 0.01 (α = 0.01 (0.05/5)). We considered *P* < 0.01 to indicate statistical significance, while 0.01 ≤ *P* < 0.05 indicated suggestive significance. Three methods, including inverse variance weighted (IVW), weighted median (WM) method and MR-Egger regression analysis, were used for MR analysis [13]. The random effects IVW method was the main MR method in this study. The IVW estimate can be acquired by an IVW meta-analysis of the ratio estimates for the individual variants. Considering that traditional IVW-MR methods are susceptible to the effects of imbalanced level pleiotropy. Therefore, we used additional MR methods, including WM and MR Egger. The WM method may provide robust estimates, even if only half of SNPs meet the requirements of valid instruments. The MR Egger method can identify and control biases caused by directional pleiotropy. Even if all variants are ineffective, as long as the association between individual variants and exposures are independent of the corresponding pleiotropic effects, the MR Egger method will still produce effective estimates. The *F* statistics of the remaining SNPs were acquired by the following formula: *F* = R^2^ × (N–k–1)/((1–R^2^) × k), where R^2^ = 2 × *β*^2^ × (1–EAF) × EAF, N is the sample size of PIGF, k is the number of SNPs, *β* is the estimate of genetic effect on PIGF, and EAF is the frequency of the effect allele. The SNPs with an *F* statistic >10 was considered strong IVs of PIGF [36].

### Sensitivity analysis

Finally, we further performed a sensitivity analysis. Cochran’s *Q* statistic was used to test heterogeneity, and *P* < 0.05 was considered statistically significant. MR-Egger regression (intercept term) was used to test pleiotropy, and *P* < 0.05 was considered statistically significant. In addition, to assess whether the MR estimates could be driven by a single SNP with significant level pleiotropy, we conducted a leave-one-out test. We performed MR analyses separately for each outcome database and then pooled effect values for atherosclerosis outcomes at different sites using a random-effects model [14].

### Availability of data and material

All data used in the present study are based on publicly available summary data from the GWAS databases. Data generated during this study are available from the corresponding author on reasonable request.

## Supplementary Materials

Supplementary Figures

Supplementary Tables
