# Supplementary material for: Causal association between placental growth factor and coronary heart disease: a Mendelian randomization study
Source: Aging (Albany NY). 2023 Oct 2;15(19):10117–32. doi: 10.18632/aging.205061 (PMC10599727; doi:10.18632/aging.205061)
Supplement: Supplementary Figures [file aging-15-205061-s001.pdf]

SUPPLEMENTARY FIGURES

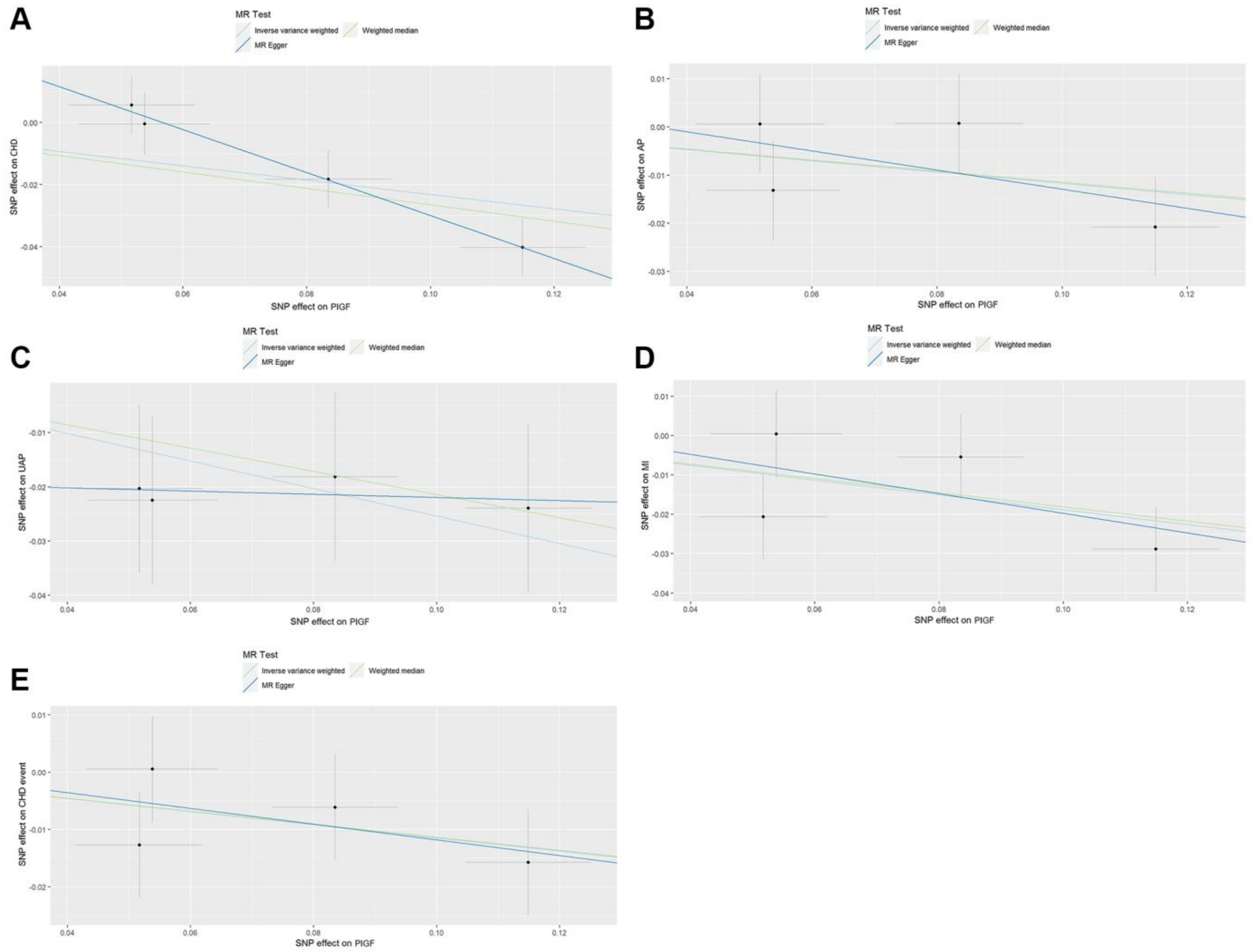

Supplementary Figure 1. Scatter plot for the effect of PlGF level on the risk of CHD. (**A**) CHD; (**B**) AP; (**C**) UAP; (**D**) MI; (**E**) CHD event. Abbreviations: CHD: coronary artery disease; AP: angina pectoris; UAP: unstable angina pectoris; MI: myocardial infarction.

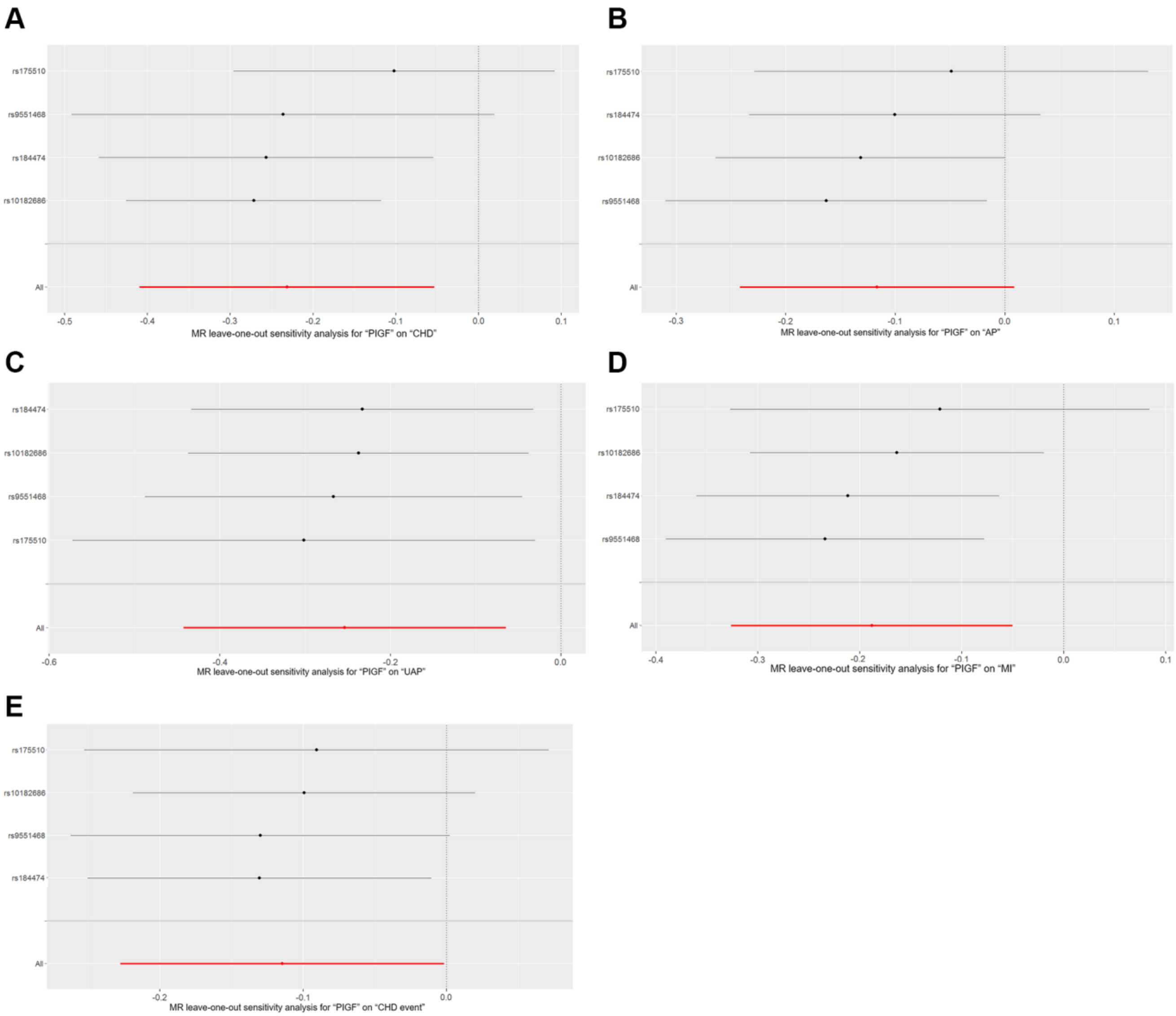

Supplementary Figure 2. The leave-one-out test. (**A**) CHD; (**B**) AP; (**C**) UAP; (**D**) MI; (**E**) CHD event. Abbreviations: CHD: coronary artery disease; AP: angina pectoris; UAP: unstable angina pectoris; MI: myocardial infarction.
