# Supplementary material for: Causal association between placental growth factor and coronary heart disease: a Mendelian randomization study
Source: Aging (Albany NY). 2023 Oct 2;15(19):10117–32. doi: 10.18632/aging.205061 (PMC10599727; doi:10.18632/aging.205061)
Supplement: Supplementary Tables [file aging-15-205061-s002.pdf]

SUPPLEMENTARY TABLES

Supplementary Table 1. Exposure and outcome data after coordination.

PIGF-AP						
**rsid**	**effect**	**SE**	**a1**	**a2**	**a1_freq**	***p*-value**
rs184474	−0.01317	0.010267	G	A	0.478912	0.199607
rs10182686	−0.00063	0.010258	G	A	0.457345	0.950928
rs9551468	0.000754	0.010283	G	C	0.461056	0.941542
rs175510	−0.02075	0.01024	A	G	0.461012	0.0426982
**PIGF-CHD event**						
**rsid**	**effect**	**SE**	**a1**	**a2**	**a1_freq**	***p*-value**
rs184474	0.00057	0.009242	G	A	0.479093	0.950788
rs10182686	0.012701	0.009245	G	A	0.457664	0.169485
rs9551468	−0.00612	0.009267	G	C	0.461014	0.50885
rs175510	−0.01577	0.009238	A	G	0.460982	0.0878942
**PIGF-UAP**						
**rsid**	**effect**	**SE**	**a1**	**a2**	**a1_freq**	***p*-value**
rs184474	−0.02248	0.01549	G	A	0.478905	0.146675
rs10182686	0.020331	0.015468	G	A	0.457386	0.188718
rs9551468	−0.01819	0.015503	G	C	0.460944	0.240767
rs175510	−0.02396	0.015438	A	G	0.461244	0.120733
**PIGF-MI**						
**rsid**	**effect**	**SE**	**a1**	**a2**	**a1_freq**	***p*-value**
rs184474	0.000392	0.011052	G	A	0.391746	0.97
rs10182686	0.020606	0.010768	G	A	0.467835	0.0560003
rs9551468	−0.00547	0.010791	G	C	0.53063	0.61
rs175510	−0.02884	0.01078	A	G	0.468535	0.00749998
**PIGF-CHD**						
**rsid**	**effect**	**SE**	**a1**	**a2**	**a1_freq**	***p*-value**
rs175510	−0.04026	0.009342	A	G	0.439054	1.64E-05
rs9551468	−0.01826	0.009388	G	C	0.513064	0.0517095
rs184474	−0.00037	0.009969	G	A	0.363761	0.970553
rs10182686	−0.00571	0.00937	G	A	0.430795	0.542558

Supplementary Table 2. Instrument strength of individual genetic variants.

	**F-statistics of individual SNP**	**R^2 of individual SNP**
Mean F statistics and Sum R^2	22	1.28%
rs184474	8	0.14%
rs10182686	7	0.13%
rs9551468	19	0.35%
rs175510	36	0.66%

Supplementary Table 3. The definition of CHD event.

Outcome	ICD code
Hospital discharge	ICD-10 — I20.0, I21, I22
Hospital discharge	ICD-9 — 410|4110
Hospital discharge	ICD-8 — 410|4110
Cause of death	ICD-10 — I21, I22, I23, I24, I25, I46, R96, R98
Cause of death	ICD-9 — 41 (0–4) |798
Cause of death	ICD-8 — 41 (0–4) |798
Cause of death	excluded ICD-9 — 7980A
